# Predictors of visual acuity improvement after phacoemulsification cataract surgery

**DOI:** 10.3389/fmed.2022.894541

**Published:** 2022-09-21

**Authors:** Saif Aldeen AlRyalat, Duha Atieh, Ayed AlHabashneh, Mariam Hassouneh, Rama Toukan, Renad Alawamleh, Taher Alshammari, Mohammed Abu-Ameerh

**Affiliations:** ^1^Department of Special Surgery, The University of Jordan, Amman, Jordan; ^2^Intern, University of Jordan Hospital, Amman, Jordan; ^3^Department of Special Surgery, Prince Mohammed Medical City, Al-Jouf, Saudi Arabia

**Keywords:** cataract, phacoemulsification, risk score, visual acuity, developing country

## Abstract

**Purpose:**

This study aimed to assess preoperative predictors of visual outcome after phacoemulsification cataract surgery in Jordan, a Middle Eastern country.

**Methods:**

This was a retrospective longitudinal study of adult patients who underwent phacoemulsification cataract surgery from January 2019 to July 2021. For each patient, we included only the first operated eye. We obtained pre-operative ocular history, cataract surgery complication risk based on a predesigned score, visual acuity, best correction, and best corrected visual acuity. We recorded intraoperative complications. We also obtained postoperative best corrected visual acuity and refractive error for correction after 1–3 months.

**Results:**

A total of 1,370 patients were included in this study, with a mean age of 66.39 (± 9.48). 48.4% of patients achieved visual acuity ≥ 0.8, and 72.7% achieved visual acuity ≥ 0.5. The mean visual acuity improvement after phacoemulsification cataract surgery was 0.33 (95% CI 0.31–0.35). In the regression model, significant predictors that affected visual acuity improvement included the presence of diabetic retinopathy, glaucoma, and complication risk factors (i.e., high-risk surgery).

**Conclusion:**

Predictors of visual acuity improvement vary between studies. This study was conducted in a developing country; we defined predictors of visual acuity improvement. We also provided a new preoperative phacoemulsification cataract surgery complication risk score.

## Introduction

Cataract extraction is considered one of the most beneficial procedures in medicine, with its outcome rapidly observed subjectively and objectively (1). According to the Global Health Commission on Global Eye Health report (2), cataract extraction is considered a “highly cost-effective vision-restoring intervention” in modern medicine. Cataract extraction *via* phacoemulsification surgery largely replaced older techniques with a high safety profile (3). Its main outcome is primarily measured by visual acuity improvement, which is translated by considerable gains in real-life activities and emotional and social life components (4). Despite the provided visual acuity improvement after phacoemulsification surgery, such improvement might not be sufficient to improve the quality of life of certain populations (5). Several studies tried to predict visual acuity improvement after phacoemulsification surgery and to provide preoperative risk factors for poor visual acuity improvement, which varied for different populations and countries and were generally of low-quality evidence (6–8). Most such studies were performed in developed countries, where surgical training and available technologies are more advanced than in developing countries. Studies from developing countries, including Jordan, are generally limited to small-size studies and cross-sectional designs (9), despite the high volume of cataract surgery performed. In this study, we aimed to analyze predictors of visual acuity gain after phacoemulsification cataract surgery in the major referral center in Jordan. This was the first study from Jordan to assess the outcome of phacoemulsification cataract surgery, where we included a relatively homogenous sample from Jordan’s largest tertiary referral center. We assessed preoperative predictors of visual outcome after phacoemulsification cataract surgery in a large cohort from the largest referral center in Jordan.

## Materials and methods

This was a retrospective longitudinal study for patients who underwent phacoemulsification cataract surgery at Jordan University Hospital, the largest tertiary referral hospital in Jordan. The patients were followed up for at least 3 months after surgery. We obtained institutional review board (IRB) committee approval from Jordan University Hospital IRB (IRB 5439/2021/67). Due to the retrospective data collection method, patients’ consent was waived, and the data were analyzed anonymously. The study was conducted in accordance with the latest declaration of Helsinki.

### Participants

We reviewed all phacoemulsification surgeries performed at Jordan’s largest tertiary referral center for 31 months, from January 1st, 2019, to July 30th, 2021. We included the first operated eye for patients who had both eyes operated on in the specified period to avoid correlated data analysis bias (10). We excluded patients with congenital cataracts or aged below 40 years (36 patients) and cataract surgeries done as part of pars plana vitrectomy (24 patients).

We reviewed the patient’s pre-operative clinic assessment, operative notes, and post-operative clinic visits. Each included patient had a pre-operative assessment visit, where visual acuity, refraction, anterior segment, and fundus exams were performed. Diabetic patients with diabetic retinopathy also underwent macular optical coherence tomography exams to exclude co-existent diabetic macular edema. Phacoemulsification surgery details obtained from each case’s operative note are detailed in the next section. Post-operatively, our institution’s standard regimen includes eye patching until the next day’s morning visit and movement restrictions for 3 days post-operatively. The next day, the eye patch was removed, and the eyes were examined, including visual acuity, wound leak, and intraocular pressure, along with an anterior segment exam. The postoperative regimen included topical fluoroquinolone antibiotics and topical steroid eye drops.

### Phacoemulsification surgery

All patients signed informed consent before entering the theater room. The eye undergoing surgery was marked, and dilating eye drops were applied 15 min before surgery. Intraoperatively, patients underwent topical, retrobulbar, or general anesthesia, depending on the patient’s factors. Each operator had an operative technique for dividing the nucleus and cortex aspiration. Stop and chop was the most commonly used technique. Otherwise, other steps were usually performed according to a standard protocol. The standard protocol intraoperatively after draping and scrubbing included paracentesis creation, injection of intracameral adrenaline and lidocaine, the use of trypan blue dye, cohesive viscoelastic to form the anterior chamber, standard up to 3 mm superior limbal clear corneal incision, capsulorhexis creation, nucleus division, aspiration, and cortex aspiration according to the surgeon’s training and preference, acrylic single-piece monofocal intraocular lens (IOL) injection in most patients (the IOL which was covered by insurance), viscoelastic aspiration, wound hydration, followed by subconjunctival moxifloxacin and steroid injection. No intracameral antibiotic is usually given per our institutional protocol.

All surgeries were performed either using R-Evolution Optikon (Italy), Geuder (Germany), or DORC (Germany) phacoemulsification machines.

### Cataract surgery complication risk scoring

We performed a literature review on cataract surgery risk for intraoperative complications and their associations with postoperative outcomes. Based on previous literature (5, 11–26), we identified several pre-operative factors that have the potential to increase surgery difficulty and complication risk. Further details about each risk score are provided in Supplementary Table 1. In regard to combining factors for a final risk score, previous studies varied from a dichotomous classification into high and low risk, which can be simpler and advantageous in statistical models; other studies used an ordinal classification scale from no risk, low risk, moderate risk, and high risk.

In our study, we classified cataract surgery complication risk into either high risk or low risk, where high-risk surgeries are those with any of the following pre-operative risk factors: Pseudoexfoliation or phacodenesis; proliferative diabetic retinopathy; previous vitrectomy; a 4 + dense, white, or brunescent cataract; age above 88; central or paracentral corneal opacity; previous penetrating keratoplasty or radial keratotomy; history of uveitis or synechia; and posterior polar cataract; high myopia (above −6); or high hyperopia (above + 3).

### Variables

We obtained demographic characteristics for each patient, including pre-operative medical history, ocular history, best corrected visual acuity, and refractive error for correction. We also obtained intraoperative data regarding the operator (senior resident or consultant), surgical notes, and any intra-operative complications, including posterior capsular rupture, dropped nucleus, or IOL, and the use of sutures to secure the wound. Finally, we obtained follow-up data for best corrected visual acuity and refractive error for correction after 1–3 months. Based on the operator, we classified surgeries into teaching cases done by senior ophthalmology residents under the supervision of consultants or cases done by consultants alone.

Visual acuities were measured on a standard E-chart at a 6-meter distance, with acuities measured in decimals. For visual acuities worse than 0.05, we converted counting fingers, hand motion, light perception, and no light perception into 0.014, 0.005, 0.0016, and 0.0013, respectively (27). Based on minimal important difference improvement, we further categorized visual acuity improvement into either improved by more than 0.1, 0.1 or less improvement or worsening in visual acuity (28, 29).

### Statistical analysis

We used SPSS version 26.0 (Chicago, USA) in our analysis. We used the mean (± standard deviation) to describe continuous variables. We used count (frequency) to describe other nominal variables. We performed linear regression analysis to assess predictors of visual acuity changes between pre-operative and post-operative visual acuity after phacoemulsification cataract surgery. We adopted a model-building strategy, where we first performed a univariate analysis, and then we only included in the regression analysis significant variables from the univariate analysis. For the univariate analysis, we performed an independent sample *t*-test and one-way ANOVA to analyze the mean difference between visual acuity and each nominal measurement (e.g., gender, operator, risk factors) and presented the data as a mean difference and 95% confidence interval (CI). We performed Pearson correlation to analyze the relationship between visual acuity difference and age, preoperative visual acuity, and refractive error. On univariate analysis, the following pre-operative predictors achieved a significance level above the pre-specified threshold: age (0.001), diabetic retinopathy (<0.001), pre-operative visual acuity (<0.001), spherical pre-operative refractive error (0.001), cylindrical preoperative refractive error (0.038), presence of glaucoma (0.003), history of intravitreal injections (<0.001), age-related macular degeneration (0.019), and cataract surgery complication risk (0.039). However, the following variables did not reach the threshold, including gender (0.666), a teaching case (0.936), laterality (0.789), and cylindrical axis of preoperative refractive error (0.762). We presented regression analysis results in B value and its 95% CI, along with model prediction accuracy, representing the model’s ability to explain the variance in the outcome. All the underlying assumptions were met. We adopted a *p*-value of 0.05 as a significant threshold.

## Results

A total of 1,370 patients were included in this study, with a mean age of 66.39 (± 9.48). They were 673 (49.1%) men and 698 (50.9%) women. Of the total cases, 312 (22.8%) were teaching cases. 48.4% of patients achieved visual acuity of ≥ 0.8, and 72.7% achieved visual acuity of ≥ 0.5. Table 1 details the characteristics of the included sample.

**TABLE 1:** The characteristics of included sample.

		Mean (Standard deviation)	Count	Column *N*%
Age		66.39 (9.48)		
Gender	Male		673	49.1%
	Female		698	50.9%
Operator	Consultant		1,055	77.2%
	Resident		312	22.8%
Laterality	Right		699	51.1%
	Left		669	48.9%
Cataract surgery complication risk	Low risk		1,021	74.5%
	High risk		350	25.5%
Ocular history	Diabetic retinopathy		254	18.6%
	Glaucoma		99	7.3%
	Age-related macular degeneration		39	2.8%
Pre-operative best corrected visual acuity		0.32 (0.26)		
Post-operative best corrected visual acuity		0.65 (0.32)		
Intra-operative complications	Posterior capsular rupture		146	10.6%
	Wound suturing		251	18.3%
	Dropped nucleus or IOL		10	0.7%

### Predictors of visual acuity improvement

The mean visual acuity improvement after phacoemulsification cataract surgery was 0.33 (95% CI 0.31–0.35), from a mean best corrected visual acuity preoperatively of 0.32 (*SD* 0.26) to 0.65 (*SD* 0.32) postoperatively. The regression model predicted 35.7% of the visual acuity change after cataract surgery based on pre-operative characteristics. The significant predictors that affected visual acuity improvement included the presence of diabetic retinopathy, glaucoma, and a complication risk factor (i.e., high-risk surgery). Moreover, increased pre-operative visual acuity, spherical refractive error, or cylindrical refractive error were also significant predictors of decreased visual acuity improvement after cataract surgery (Table 2).

**TABLE 2:** Predictors of visual acuity improvement.

Factor	Impact on visual acuity improvement	95.0% confidence interval		*P*-value
Presence of diabetic retinopathy	−0.095	−0.182	−0.007	0.034
Presence of glaucoma	−0.123	−0.220	−0.026	0.013
High-risk cataract surgery	−0.071	−0.138	−0.004	0.037
Each 0.1 increase in pre-operative vision	−0.0653	−0.0772	−0.0534	0.000
A dioptric increase in spherical refractive error	−0.010	−0.018	−0.002	0.011
A dioptric increase in cylindrical refractive error	−0.051	−0.081	−0.021	0.001

The model building strategy and included variables were detailed in the statistical analysis section.

### Cataract surgery complication risk factors

**FIGURE 1:**
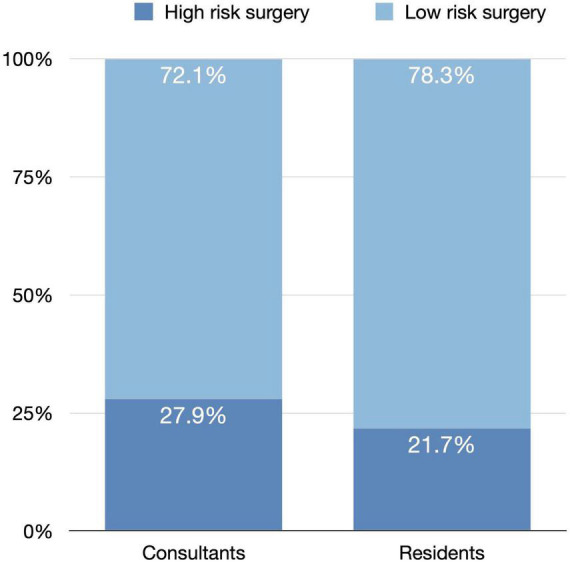
The frequency of each risk factor studies for cataract surgeries.

A total of 350 (25.5%) surgeries were high-risk surgeries. They had a total of 382 risk factors, whereas 39 surgeries had more than one risk factor. The most common risk factor was pseudoexfoliation (23.56%), followed by high myopia (22.25%) and proliferative diabetic retinopathy (19.9%). Figure 1 shows the frequency of each risk factor for cataract surgeries.

We found a significant difference in visual acuity improvement between high-risk and low-risk surgeries (*p* = 0.039), where the mean visual acuity improvement in low-risk surgeries was 0.355 (*SD* 0.31), compared to 0.301 (*SD* 0.33) for high-risk surgeries (mean difference 0.054, 95% CI 0.003–0.105). No significant difference was found in the intra-operative complication rate between both groups (*p* = 0.523).

### Teaching cases

**FIGURE 2:**
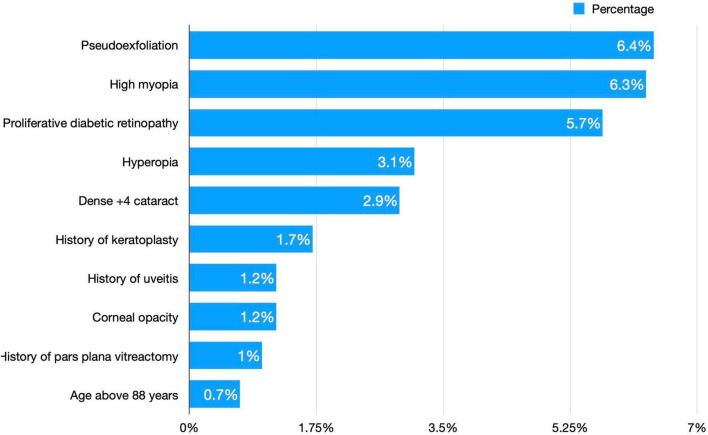
A comparison of surgery difficulty between consultants and residents who performed phacoemulsification surgeries.

Teaching cases operated by senior residents under the supervision of consultants comprised 312 (22.8%) cases. The majority of these cases were of low risk (78.8%), with only 66 (21.2%) cases of high risk compared to 283 (26.8%) non-teaching cases, a frequency that differed significantly (*p* = 0.025). No significant difference in visual acuity gains after cataract surgery (*p* = 0.940) or frequency of complications (*p* = 0.336) between teaching and non-teaching cases. Figure 2 compares consultants and residents who performed surgeries regarding surgery difficulty.

### Refractive error change after cataract surgery

Upon comparing refractive error change after cataract surgery, we found a significant difference in spherical refractive error (*p* < 0.001), with a mean increase in spherical refractive error by a mean of 2.18 (95% CI −2.74 to −1.62). No significant difference was found in cylindrical refractive errors or their axes (Table 3).

**TABLE 3:** Refractive error change after cataract surgery.

		Mean	Std. deviation	Mean difference (95% CI)	*P*-value
Spherical equivalence change	Pre-op	−0.98	1.17156	−0.23 (−0.48 to 0.02)	0.075
	Post-op	-0.75	1.32752		
Spherical refractive error change	Pre-op	−1.99	3.71822	−2.18 (−2.74 to −1.62)	<0.001
	Post-op	0.19	1.00916		
Cylindrical refractive error change	Pre-op	1.55	1.08636	−0.09 (−0.36 to 0.18)	0.514
	Post-op	1.64	1.31116		
Cylinder axis change	Pre-op	92.64	38.831	1.25 (−6.41 to 8.92)	0.746
	Post-op	91.39	33.733		

### Clinically meaningful visual acuity change

After categorizing patients into three categories, we found that most patients had an improvement of > 0.1 in visual acuity (69.4%), while 20% of patients had 0.1 or less visual acuity improvement, and only 10.6% had a worsening in visual acuity. Baseline visual acuity was significantly associated with each category of visual acuity improvement (*p* < 0.001). In addition, the visual acuity worsening group had a higher cataract surgery complication risk. Table 4 compares the mean baseline visual acuity and complication risk among the three categories.

**TABLE 4:** Comparison between best corrected visual acuity (BCVA) improvement by > 0.1, ≤0.1, and worsening in terms of mean baseline visual acuity and complication risk among the three categories.

	> 0.1 BCVA improvement	≤ 1 BCVA improvement	BCVA worsening	*P*-value
Mean (95% CI) baseline visual acuity	0.28 (95% CI 0.29–0.32)	0.34 (95% CI 0.28–0.39)	0.42 (95% CI 0.35–0.49)	<0.001
High risk for complication	25.7%	35.4%	41.7%	0.002

## Discussion

This study was the largest to define predictors of visual acuity improvement after phacoemulsification cataract surgery. The mean improvement expected after phacoemulsification cataract surgery was 0.33 (95% CI 0.31–0.35); this magnitude of improvement would decrease if the eye had glaucoma, diabetic retinopathy, pre-operative complication risk factors, higher pre-operative visual acuity, or refractive error. We also performed a literature review to find factors that increase the risk of surgical complications, and we classified phacoemulsification into high- and low-risk surgeries accordingly. We found that surgeries classified as high-risk had significantly lower visual acuity improvement compared to low-risk surgeries. Almost 23% of included cases were teaching cases operated by senior ophthalmology residents, and we did not find a higher complication rate or worse visual acuity in teaching cases. Regarding refractive error change after phacoemulsification cataract surgery, we found an improvement in spherical error. A European Registry of Quality Outcomes for Cataract and Refractive Surgery study found that ocular comorbidities were the most important predictor of visual acuity improvement, where ocular comorbidities included macular degeneration, glaucoma, diabetic retinopathy, and amblyopia, among others (7). Another US-based study also found pre-operative comorbidities to be predictors of poor visual acuity, which included diabetes mellitus, chronic pulmonary disease, and age-related macular degeneration (30).

Among the factors that affect the outcome of cataract surgery is the difficulty and complexity of the surgery itself, which can be predicted by preoperative factors (31). The complexity of cataract surgery was one of the most commonly appearing predictors of poorer visual acuity improvement (13, 32). Considering preoperative risk scoring in surgery, decision-making and planning should also be included during the surgery decision-making process (5). Studies used different scores to classify surgeries into high-risk (aka. complex surgery) and low-risk surgeries. In the study by Lundström et al. complex surgery is defined by the presence of previous vitrectomy, previous corneal refractive surgery, miosis, white/brown cataract, corneal opacities, pseudoexfoliation, and others (7). Another negative predictor factor of visual acuity improvement was glaucoma. The relationship between cataract extraction and glaucoma is complex. Although it has been established that cataract extraction has a beneficial intraocular pressure lowering effect and improves the quality of life (33, 34), phacoemulsification cataract extraction surgery might sometimes be challenging in these patients. Patients with glaucoma usually also have other ocular co-morbidities, both diagnosed and undiagnosed, along with frequent topical medication use (35, 36). After surgery, glaucoma patients experience increased intraocular pressure, severe corneal edema, endothelial cell damage, and poor vision (37, 38). A study performed on a European registry of 15 European countries found that preoperative ocular co-morbidity was the strongest negative predictor for visual outcome, where comorbidities included glaucoma and other retinal diseases (7). A previous study in several African developing countries found that pre-operative refractive error was the leading cause of poor visual outcomes (39). Consultants operated at a higher frequency of high-risk surgeries compared to residents, a finding also found in a UK-based national study (40). A recent systematic review found that the previous history of intravitreal injection can be regarded as a risk factor for PCR and should be considered when planning cataract surgery. However, the magnitude of this risk is generally small (41). The complexity of preoperative risk score discussion increases when we consider protective factors that might decrease surgery difficulty or complication rate (42), which should be considered in future studies.

In our study, no significant difference in complication rates was found between teaching cases operated by residents and non-teaching cases operated by specialists. Our results were consistent with previous studies done in other countries, including the USA (43), the UK (40), Canada (44), and Australia (45). On the other hand, a recent study on surgeries performed in Europe found higher complication rates for surgeries performed by residents (46). Higher complication rates for residents were also found in studies done in Hungary (47). It is important to note that these studies differed in settings, countries, and teaching methods. A future review investigating surgical factors and teaching methods might reveal the reason behind these differences. While we did not measure the duration of surgery, a previous study found that the duration of surgery significantly differed according to experience, with the longest duration for trainees and the shortest duration for experienced specialists (48).

Our study is the first in Jordan and the Middle East to assess the visual outcome and predictors of visual acuity in a large cohort; its main limitation is the use of a retrospective design for data collected from university hospital-based ophthalmology clinics. As a result, we could not include certain factors that may be considered pre-operative risk factors due to under-reporting by patients’ records.

## Conclusion

In our cohort from Jordan, a developing country, we found that the mean improvement expected after phacoemulsification cataract surgery was 0.33 (95% CI 0.31–0.35), where the mean best corrected visual acuity after cataract surgery was 0.65 (*SD* 0.32) postoperatively, which is above the limit for driving in most countries. The majority of patients had visual acuity improvement in more than one line. Patients with higher baseline visual acuity would be expected to improve less than patients with lower baseline visual acuity. Poor visual acuity improvement predictors include glaucoma, diabetic retinopathy, pre-operative complication risk factors, higher pre-operative visual acuity, and refractive error. We provided a literature-based new preoperative phacoemulsification cataract surgery complication risk score.

## What was known

• Phacoemulsification has revolutionized the management of cataracts in recent years. However, there has been wide variation in its outcome and predictors of outcome between different studies in different countries.
• Most such studies were performed in developed countries, where surgical training and available technologies are more advanced than in developing countries.

## What this paper adds

• Our study is the first in Jordan, a developing country, and the Middle East to assess the visual outcome and predictors of visual acuity in a large cohort.
• We also provided a literature-based new preoperative phacoemulsification cataract surgery complication risk score.

## Data availability statement

The raw data supporting the conclusions of this article will be made available by the authors, without undue reservation.

## Ethics statement

The studies involving human participants were reviewed and approved by we obtained institutional review board (IRB) committee approval from Jordan University Hospital IRB (IRB 5439/2021/67). Due to the retrospective data collection method, patients’ consent was waived, and the data were analyzed anonymously. The study was conducted in accordance with the latest declaration of Helsinki.

## Author contributions

SA, DA, and MA-A contributed to research conception, protocol development, manuscript writing, and data analysis. AA, MH, RT, and RA contributed to data collection and manuscript writing. TA contributed to research conception and manuscript writing. All authors approved final manuscript.
