# Supplementary material for: Predictors of visual acuity improvement after phacoemulsification cataract surgery
Source: Front Med (Lausanne). 2022 Sep 21;9:894541. doi: 10.3389/fmed.2022.894541 (PMC9532505; doi:10.3389/fmed.2022.894541)
Supplement: Supplementary file 1 [file Table_1.pdf]

Supplementary Table 1. Comparison in pre-operative phacoemulsification cataract surgery risk scoring

Study	Year	New or used a previous one	Factors included in the risk calculation	Scoring system
Muhtaseb et al. (1)	2004	New	High risk (3 points each)<br>• Dense/total/white or brunescent cataract<br>• Pseudoexfoliation<br>• Phacodonesis<br>Low risk (1 point each)<br>• Previous vitrectomy<br>• Corneal scarring<br>• Small pupil (3 mm)<br>• Shallow anterior chamber (depth,2.5 mm)<br>• Age.88 years<br>• High ametropia (.6 D of myopia or hyperopia)<br>• Posterior capsule plaque<br>• Posterior polar cataract<br>• Miscellaneous risks assessed by the surgeon (eg, poor position of eye/patient)	Four risk groups:<br>group 1 (no added risk) 0 points; group 2 (low risk) 1–2 points; group 3 (moderate risk) 3–5 points;<br>group 4 (high risk) 6 points or more
Kaur et al. (2)	2020	New Literature review!!	• hard sclerotic nucleus<br>• white cataract<br>• pseudoexfoliation<br>• posterior polar cataract<br>• subluxated cataract<br>• small pupil	Just identified risk factors
Blomquist et al. (3)	2011	New	On univariate analysis<br>• older age<br>• poor preoperative corrected distance visual acuity (CDVA)<br>• left eye<br>• history of trauma<br>• prior pars plana vitrectomy<br>• dementia<br>• phacodonesis<br>• zonule dehiscence<br>• posterior polar cataract<br>• white/mature cataract<br>• dense nuclear sclerotic cataract<br>• poor red reflex<br>On multivariate analysis<br>• older age<br>• poor preoperative CDVA<br>• left eye<br>• prior pars plana vitrectomy<br>• dementia<br>• zonule dehiscence	Just identified risk factors
Mylona et al. (4)	2020	New	high-risk (9 points each)<br>• Phacodonesis<br>• dense/total/ white or brunescent cataract<br>• pseudoexfoliation<br>moderate-risk (3 points each)<br>• Glaucoma<br>• poor cooperation	3 risk groups and a no-risk group 1:<br>-Low risk group : 1 point<br>-Moderate-risk group : sum of 3 points<br>-High-risk group : sum of 9 points

			• corneal scarring<br>• shallow anterior chamber<br>low-risk (1 point each)<br>• deep orbit<br>• ametropia<br>• vitrectomy<br>• loss of 1 eye<br>• small pupil<br>• age younger than 50 years or older than 85 years<br>• diabetes<br>• a1-antagonist use	
Zetterberg et al.(5)	2020	New	• best-corrected visual acuity (BCVA) ≤0.1<br>• pseudoexfoliation (PEX)<br>• sight-threatening ocular comorbidity other than age-related macular degeneration (AMD), diabetic retinopathy, glaucoma, or cornea guttata ;<br>• use of Trypan blue<br>• mechanical pupil dilation<br>• iris hooks at the rhexis margin	A composite risk score using odds ratio
Ergun et al. (6)	2018	New	• Age (60–69/B80)<br>• male gender<br>• pupil size B3 mm<br>• mature- brunescent cataract<br>• anterior chamber depth \2.5 mm posterior polar cataract<br>• diabetic retinopathy<br>• coronary artery disease<br>• surgeon factor (junior resident/senior resident; senior resident/specialist in ophthalmology; junior resident/specialist in ophthalmology)	A composite risk score using odds ratio
Nderitu et al. (7)	2018	New	Factors associated with PCR<br>• age<br>• male sex<br>• systemic a-blocker use<br>• glaucoma<br>• diabetic retinopathy<br>• axial length (AL)<br>• small pupil less than 4.0 mm<br>• poor positioning<br>• white cataract<br>• no fundal view<br>• pseudoexfoliation or phacodonesis<br>Patient-specific factors<br>• significant hearing impairment<br>• previous iridectomy<br>• previous cystoid macula edema [CME] or retinal vein occlusion<br>• guttata, or increased corneal thickness<br>• anterior chamber depth [!2.5 mm], or spherical equivalent more than 5.0 diopters<br>• permanent poor vision in the unoperated eye	5 complexity groups:<br>-Group 1: score 0 to 1<br>-Group 2: score 2 to 3<br>-Group 3: score 4 to 7<br>-Group 4: score 8 to 9<br>-Group 5: score 10 or more<br>(Each variable is attributed an approximate proportional score)
Hashemi et al. (8)	2013	New	On univariate analysis<br>• diabetes mellitus<br>• shallow anterior chamber	Just identified risk factors

			• absence of supervision by a faculty member<br>• larger capsulorhexis<br>• anterior capsule tear<br>• longer effective phacoemulsification time (EPT)<br>On multivariate analysis<br>• anterior capsule tear<br>• longer EPT<br>• lack of supervision by attending physicians	
Rutar et al. (9)	2009	New	On univariate analysis<br>• challenging case (mature cataracts and potential zonular pathology were most strongly associated with major complications)<br>• wound type<br>• phacoemulsification technique preoperative visual acuity<br>On multivariate analysis<br>• challenging case	Just identified risk factors
Lomi et al. (10)	2015	New	• Increase in success rate was seen with increase in semester and number of surgeries performed.<br>Systemic and ocular features of patients as well as type of machine (longitudinal versus torsional longitudinal) had no significant association in terms of complication rate.	Just identified risk factors
Gharaei et al. (11)	2015	New	• poor red reflex<br>• pseudoexfoliation<br>• poor pupil dilation<br>• corneal clouding<br>• dense cataract<br>• shallow anterior chamber<br>• advanced age	Just identified risk factors
Narendran et al. (12)	2009	New	• increasing age<br>• male gender<br>• presence of glaucoma<br>• diabetic retinopathy<br>• brunescent/white cataract<br>• no fundal view/vitreous opacities<br>• pseudo-exfoliation/phacodonesis<br>• reducing pupil size<br>• axial length > or = 26.0 mm<br>• the use of the alpha-blocker doxazosin<br>• inability to lie flat<br>• trainee surgeons performing operations	A composite risk score using odds ratio
Saifee et al.(13)	2020	New	• pseudoexfoliation<br>• proliferative diabetic retinopathy<br>• previous vitrectomy<br>• a 4+ dense, white, or brunescent cataract<br>• current tamsulosin use<br>• preexisting zonular dialysis<br>• intraoperative use of iris hooks<br>• intraoperative use of a pupillary expansion device	3 risk groups:<br>low risk (0 risk factors)<br>intermediate risk (1 risk factor)<br>high risk (≥2 risk factors)
Habib et al.(14)	2005	New	General condition<br>• Unable to lie flat (spinal deformity, asthma, heart failure)<br>• Severe anxiety	A cumulative risk score (Total scores could range from 1 in simple cases to a maximum possible score of

			• Head tremors<br>Ocular history<br>• Previous angle closure glaucoma<br>• History of complications in fellow eye<br>• Previous vitrectomy plus or minus silicone oil<br>Ocular co-morbidity<br>• Corneal cloudiness<br>• Shallow anterior chamber<br>• Poor pupillary dilatation and/or posterior synaechiae<br>• Pseudoexfoliation<br>• Weak zonules<br>• High hypermetropia (axial length ,20)<br>• High myopia (axial length .27)<br>Cataract density<br>• Nuclear density grade 1–2<br>• Nuclear density grade 3<br>• Mature brunescent cataract	13 points in difficult cases)<br><br>(Each variable is attributed an approximate proportional score)
Najjar and Awwad (15)	2003	New	• Age<br>• type of anesthesia administered<br>• density of the cataract<br>• frontal bossing or sunken globes<br>• pseudoexfoliation<br>• extremes of refraction<br>• a history of glaucoma or uveitis<br>• previous intraocular surgery<br>• a history of complications in the fellow eye<br>• poor pupil dilation<br>• shallow anterior chamber<br>• corneal cloudiness<br>• poor red reflex	A cumulative risk score (The risk score can vary from 2 to 25, with 2 given to the simplest case and 25 to the most complex)<br><br>(Each variable is attributed an approximate proportional score)

Reference:

1. Muhtaseb M, Kalhoro A, Ionides A. A system for preoperative stratification of cataract patients according to risk of intraoperative complications: a prospective analysis of 1441 cases. *Br J Ophthalmol* (2004) 88:1242. doi: 10.1136/bjo.2004.046003

2. Kaur M, Bhai N, Titiyal JS. Risk factors for complications during phacoemulsification cataract surgery. *Expert Rev Ophthalmol* (2020) 15:303–312. doi: 10.1080/17469899.2020.1806715

3. Blomquist PH, Morales ME, Tong L, Ahn C. Risk factors for vitreous complications in resident-performed phacoemulsification surgery. *J Cataract Refract Surg* (2012) 38:208–214. doi: 10.1016/j.jcrs.2011.10.001

4. Mylona I, Dermenoudi M, Glynatsis M, Ziakas N, Tsinopoulos I. Development of a reliable preoperative risk stratification system for phacoemulsification. *J Cataract Refract Surg* (2020) 46:1132–1137. doi: 10.1097/j.jcrs.0000000000000223

5. Zetterberg M, Kugelberg M, Nilsson I, Lundström M, Behndig A, Montan P. A Composite Risk Score for Capsule Complications Based on Data from the Swedish National Cataract Register: Relation to Surgery Volumes. *Ophthalmology* (2021) 128:364–371. doi: 10.1016/j.ophtha.2020.07.033

6. Ergun ŞB, Kocamış Sİ, Çakmak HB, Çağıl N. The evaluation of the risk factors for capsular complications in phacoemulsification. *Int Ophthalmol* (2018) 38:1851–1861. doi: 10.1007/s10792-017-0667-3

7. Nderitu P, Ursell P. Updated cataract surgery complexity stratification score for trainee ophthalmic surgeons. *J Cataract Refract Surg* (2018) 44:709–717. doi: 10.1016/j.jcrs.2018.04.036

8. Hashemi H, Mohammadpour M, Jabbarvand M, Nezamdoost Z, Ghadimi H. Incidence of and risk factors for vitreous loss in resident-performed phacoemulsification surgery. *J Cataract Refract Surg* (2013) 39:1377–1382. doi: 10.1016/j.jcrs.2013.03.028

9. Rutar T, Porco TC, Naseri A. Risk Factors for Intraoperative Complications in Resident-Performed Phacoemulsification Surgery. *Ophthalmology* (2009) 116:431–436. doi: 10.1016/j.ophtha.2008.10.028

10. Lomi N, Sharma R, Khokhar S, Dada T, Vanathi M, Agarwal T. Risk factors for intra-operative complications during phacoemulsification performed by residents. *Int Ophthalmol* (2016) 36:401–406. doi: 10.1007/s10792-015-0146-7

11. Gharaei H, Sedaghat MR, Banan S. Evaluation of the Correspondence between Preoperative Risk Factors and Intraoperative Complications in Resident-Performed Phacoemulsification. *J Patient Saf Qual Improv* (2015) 3:273–276. doi: 10.22038/psj.2015.5243

12. Narendran N, Jaycock P, Johnston RL, Taylor H, Adams M, Tole DM, Asaria RH, Galloway P, Sparrow JM. The Cataract National Dataset electronic multicentre audit of 55,567 operations: risk stratification for posterior capsule rupture and vitreous loss. *Eye Lond Engl* (2009) 23:31–37. doi: 10.1038/sj.eye.6703049

13. Saifee M, Zhu I, Lin Y, Oldenburg CE, Ramanathan S. Effect of full-time vs volunteer faculty supervision on resident cataract surgery complications. *J Cataract Refract Surg* (2020) 46:700–704. doi: 10.1097/j.jcrs.0000000000000145

14. Habib MS, Bunce CV, Fraser SG. The role of case mix in the relation of volume and outcome in phacoemulsification. *Br J Ophthalmol* (2005) 89:1143–1146. doi: 10.1136/bjo.2005.070235

15. Najjar DM, Awwad ST. Cataract surgery risk score for residents and beginning surgeons. *J Cataract Refract Surg* (2003) 29:2036–2037. doi: 10.1016/j.jcrs.2003.08.004
